# ^1^H NMR-Based Isolation of Anti-Inflammatory 9,11-Secosteroids from the Octocoral *Sinularia leptoclados*

**DOI:** 10.3390/md18050271

**Published:** 2020-05-21

**Authors:** Yu-Chia Chang, Kuei-Hung Lai, Sunil Kumar, Po-Jen Chen, Yi-Hsuan Wu, Ching-Long Lai, Hsi-Lung Hsieh, Ping-Jyun Sung, Tsong-Long Hwang

**Affiliations:** 1 Research Center for Chinese Herbal Medicine, Graduate Institute of Healthy Industry Technology, College of Human Ecology, Chang Gung University of Science and Technology, Taoyuan 333324, Taiwan; jay0404@gmail.com (Y.-C.C.); mos19880822@gmail.com (K.-H.L.); yhwu03@mail.cgust.edu.tw (Y.-H.W.); dinolai@mail.cgust.edu.tw (C.-L.L.); 2 Chinese Herbal Medicine Research Team, Healthy Aging Research Center, Chang Gung University, Taoyuan 333323, Taiwan; suniliftm1982@gmail.com; 3 Department of Cosmetic Science, Providence University, Taichung 433303, Taiwan; litlep@hotmail.com; 4 Department of Nursing, Division of Basic Medical Sciences, Chang Gung University of Science and Technology, Taoyuan 333324, Taiwan; 5 Department of Neurology, Chang Gung Memorial Hospital, Taoyuan 333423, Taiwan; 6 National Museum of Marine Biology and Aquarium, Pingtung 944401, Taiwan; 7 Graduate Institute of Marine Biology, National Dong Hwa University, Pingtung 944401, Taiwan; 8 Chinese Medicine Research and Development Center, China Medical University Hospital, Taichung 404394, Taiwan; 9 Graduate Institute of Natural Products, College of Medicine, Chang Gung University, Taoyuan 333323, Taiwan; 10 Department of Anaesthesiology, Chang Gung Memorial Hospital, Taoyuan 333423, Taiwan; 11 Department of Chemical Engineering, Ming Chi University of Technology, New Taipei City 243303, Taiwan

**Keywords:** *Sinularia leptoclados*, secosteroids, antineutrophilic inflammation, elastase, superoxide anion

## Abstract

Octocoral *Sinularia leptoclados* has been identified as a source of bioactive 9,11-secosteroids. This study adopted a targeted isolation approach to the discovery and analysis of five 9,11-secosteroids, including two novel compounds named sinleptosterols A (**1**) and B (**2**) as well as five known analogues (8*α*H-3*β*,11-dihydroxy-24-methylene-9,11-secocholest-5-en-9-one (**3**), 8*β*H-3*β*,11-dihydroxy-24-methylene-9,11-secocholest-5-en-9-one (**4**), leptosterol A (**5**), (24*S*)-3*β*,11-dihydroxy-24-methyl-9,11-secocholest-5-en-9-one (**6**), and 3*β*,11-dihydroxy-9,11-secogorgost-5-en-9-one (**7**)) in terms of ^1^H-NMR patterns and potency against neutrophilic inflammation. The structure of secosteroids **1** and **2** was deduced from general spectroscopic analysis and an examination of NMR spectra. Among the above-mentioned isolates, compound **4** had the most pronounced effect in inhibiting elastase release and superoxide anion generation, with the IC_50_ values of 2.96 and 1.63 μM, respectively.

## 1. Introduction

Inflammation is one aspect of the regular host reaction to injury or infection caused by toxic chemicals, dead cells, pathogens, irritants, or allergens. The important role of neutrophils in a variety of infectious and inflammatory diseases makes them an attractive target for therapeutic interventions [1]. Numerous herbs and plant-derived compounds have been found to alleviate inflammation [2]; however, very few sources have been identified in marine environments.

Soft corals are reported to produce a variety of secondary metabolites with diverse pharmacological activities. Most of the metabolites from soft corals are sesquiterpenes, diterpenes, and steroids [3]. The octocoral *Sinularia leptoclados* belongs to the order Alcyonacea, which has been shown to produce a remarkable diversity of steroids in large quantities [4,5,6]. The 9,11-secosteroids found in marine invertebrates such as sponges, corals, ascidian, and mollusk can be structurally characterized by the C-9/11 oxidative cleavage of the C-ring [7,8]. The potent inhibitory effects of 9,11-secosteroids toward neutrophilic inflammation [9,10,11] motivated the current study of chemical compositions with this structural feature.

**Figure 1:**
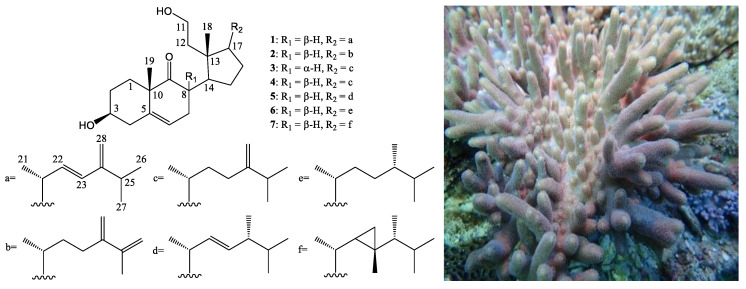
The structures of 9,11-secosteroids sinleptosterols A (**1**) and B (**2**), 8*α*H-3*β*,11-dihydroxy-24-methylene-9,11-secocholest-5-en-9-one (**3**), 8*β*H-3*β*,11-dihydroxy-24-methylene-9,11-secocholest-5-en-9-one (**4**), leptosterol A (**5**), (24*S*)-3*β*,11-dihydroxy-24-methyl-9,11-secocholest-5-en-9-one (**6**), and 3*β*,11-dihydroxy-9,11-secogorgost-5-en-9-one (**7**), and a picture of *S. leptoclados*.

In this study, an ethyl acetate (EtOAc) extract of *S. leptoclados* displayed notable anti-inflammatory activity (superoxide anion generation: IC_50_ 3.97 μg/mL) following the isolated targeting of 9,11-secosteroids based on ^1^H-NMR and bioassay information. Column chromatography revealed two novel 9,11-secosteroids (sinleptosterols A (**1**) and B (**2**)) in addition to five known metabolites **3**–**7** (Figure 1). Most of the 9,11-secosterols isolated in this study displayed notable inhibitory effects on *N*-formyl-methionyl-leucyl-phenylalanine/cytochalasin B (fMLP/CB)-induced superoxide anion generation and elastase release.

## 2. Results and Discussion

### 2.1. ^1^H NMR-Based Isolation of Anti-Inflammatory 9,11-Secosteroids

**Figure 2:**
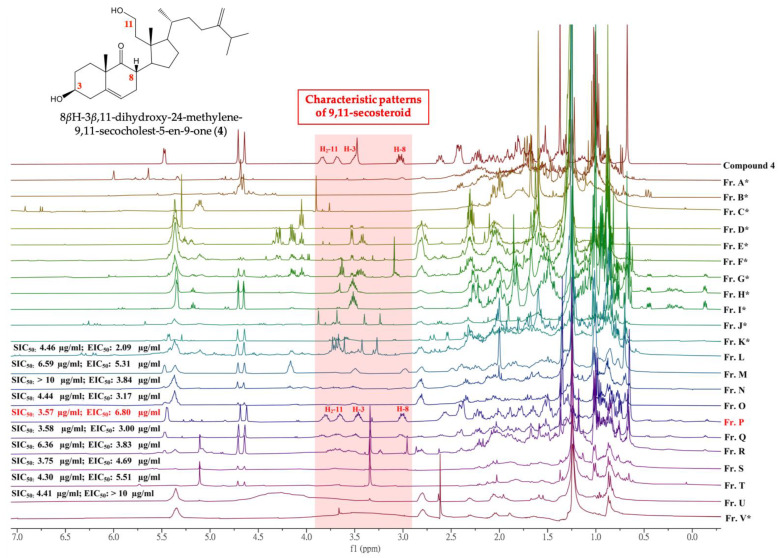
^1^H-NMR spectra of fractions derived from the EtOAc extract of octocoral *S. leptoclados* extract as well as their corresponding inhibitory effects on superoxide anion generation (SIC_50_) and elastase release (EIC_50_) in *N*-Formylmethionyl-leucyl-phenylalanine (fMLF)/cytochalasin B (CB)-induced human neutrophils. * Both SIC_50_ and EIC_50_ are over 10 μg/mL.

Primary silica gel chromatographic fractionation was used to probe anti-inflammatory 9,11-secosteroids within the organic extract from *S. leptoclados*. Comprehensive chemical and biological profiles of all fractions (fractions A–V) were then constructed through ^1^H-NMR analysis and an examination of anti-inflammatory activity (Figure 2). Fraction P was selected for subsequent analysis due to its characteristic 9,11-secosteroidal ^1^H-NMR patterns (δ_H_ 3.51, 1H, m, H-3; 3.03, 1H, ddd, *J* = 12.4, 12.8, 6.8, H-8; 3.86, 1H, m; 3.74, 1H, m, H_2_-11) and potent anti-inflammatory activities (superoxide anion generation: IC_50_ 3.57 μg/mL; elastase release: IC_50_ 6.80 μg/mL). Consecutive column chromatographic processes (reverse phase) resulted in the isolation of two novel 9,11-secosteroids, (sinleptosterols A (**1**) and B (**2**)) as well as 8*α*H-3*β*,11-dihydroxy-24-methylene-9,11-secocholest-5-en-9-one (**3**) [12], 8*β*H-3*β*,11-dihydroxy-24-methylene-9,11-secocholest-5-en-9-one (**4**) [12], leptosterol A (**5**) [5], (24*S*)-3*β*,11-dihydroxy-24-methyl-9,11-secocholest-5-en-9-one (**6**) [5], and 3*β*,11-dihydroxy-9,11- secogorgost-5-en-9-one (**7**) [12] (Figure 1).

### 2.2. Chemical Identification of 9,11-Secosterols

Compound **1** was obtained as a colorless oil. Positive mode high resolution electrospray ionization mass spectrum ((+)-HRESIMS)) of **1** revealed a sodiated adduct ion peak at *m*/*z* 451.31811, which established the molecular formula C_28_H_44_O_3_ (calcd. for C_28_H_44_O_3_ + Na, 451.31827), indicating seven degrees of unsaturation. IR absorption observed at 3369, 2958, and 1708 cm^−1^ suggested the presence of hydroxy, alkene, and ketonic groups (see Supplementary Materials Figure S2). The ^13^C and distortionless enhancement by polarization transfer (DEPT) spectroscopic data revealed 28 carbon signals in this compound, (Table 1), including five methyls, eight sp^3^ methylenes (including an oxymethylene), one sp^2^ methylene, six sp^3^ methines (including one oxymethine), two sp^3^ quaternary carbons, three sp^2^ methines, and three sp^2^ quaternary carbons (including two olefin carbons and one ketonic carbonyl) (see Supplementary Materials Figure S5). It was found that the quaternary carbon signal at δ_C_ 217.5 (C-9) and the proton shifts at δ_H_ 1.40 (s, H_3_-19), 3.03 (ddd, *J* = 12.4, 12.4, 6.8 Hz, H-8), 3.51 (m, H-3), and 5.47 (d, *J* = 5.6 Hz, H-6) were similar to those of 3-hydroxy-9,11-seco-9- oxosterols (with a 5,6-double bond). A disubstituted alkene was recognized within the carbon signals at δ_C_ 134.6 (CH-22) and 130.2 (CH-23), and was further confirmed by two olefin proton signals at δ_H_ 5.65 (1H, dd, *J* = 16.0, 7.2 Hz, H-22) and 5.93 (1H, d, *J* = 16.0 Hz, H-23) (Table 1). Three methyl doublets at δ_H_ 1.08 (3H, *J* = 6.4 Hz), 1.06 (3H, *J* = 6.8 Hz), and 1.07 (3H, *J* = 6.8 Hz) can respectively be attributed to the Me-21, Me-26, and Me-27 methyl groups. Two sharp methyl singlets for H_3_-18 and H_3_-19 respectively appeared at δ_H_ 0.69 (3H, s) and 1.40 (3H, s). Taken together, these findings identify compound **1** as a tricyclic compound.

^1^H NMR coupling information within the correlation spectroscopy (COSY) data of **1** enabled identification of separate spin systems for H_2_-1/H_2_-2/H-3/H_2_-4, H_2_-11/H_2_-12, H-6/H_2_-7/H-8/H-14/H_2_-15/H_2_-16/H-17/H-20/H-22/H-23, H-20/H_3_-21, and H-25/H_3_-26/H_3_-27 (Table 1), which were experimentally assembled with the assistance of a heteronuclear multiple bond correlation (HMBC). Key HMBC between protons and quaternary carbons of **1**, such as H-4, H_2_-7, H_3_-19/C-5; H-8, H-14, H_3_-19/C-9; H-6, H_3_-19/C-10; H_2_-12, H_3_-18/C-13 and H-22, H-23, H-25, H_3_-26, H_3_-27, H_2_-28/C-24, permitted elucidation of the carbon skeleton of **1** (Table 1).

**Figure 3:**
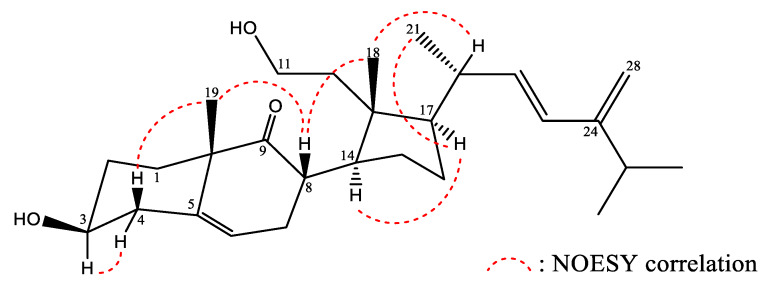
Selected NOESY correlations observed for **1**.

The relative stereochemistry of **1** was explained in terms of correlations observed in a nuclear Overhauser effect spectroscopy (NOESY) experiment, and through a comparison of NMR data between **1** and known secosterol **5**. The results suggest that these two compounds possess the same 9,11-secosterol skeleton as well as the same core A-, B-, and D-rings [5]. The configurations at C-3, C-8, C-10, C-13, C-14, and C-17 in **1** were found to be identical to those of **5**. Key NOESY correlations for **1** displayed interactions between H-8/H_3_-18 and H-8/H_3_-19. Thus, H-8 should be located on the β-face (Figure 3). A large coupling constant (*J* = 16.0 Hz) indicated a *trans* relationship between H-22 and H-23. All these data allowed to identify compound (**1**) as depicted in Figure 1 and it was named sinleptosterol A.

The molecular formula of compound (**2**) was the same as that of **1** (C_28_H_44_O_3_), as determined by (+)-HRESIMS at *m*/*z* 451.3239 (calcd. for C_28_H_44_O_3_ + Na, 451.3188), with seven degrees of unsaturation. Its IR bands revealed the presence of hydroxy (3406 cm^−1^) and ketone (1708 cm^−1^) groups (see Supplementary Materials Figure S11). On the basis of the above analysis and a comparison of one-dimensional and two-dimensional NMR experiments on **2** (Table 2), the core structural systems of secosterol **2** were established. It was found that the ^1^H and ^13^C NMR chemical shifts of **2** (including coupling patterns and coupling constants) resembled those of **1**; however, the signals corresponding to the disubstituted alkene between C-22/23 in **1** were replaced by aliphatic methylenes in **2**, and one of the methyl groups at C-25 in **1** (Me-26) was replaced by an exocyclic carbon-carbon bond in **2**.

The observed HMBC correlations fully supported the locations of the functional groups. An olefinic bond was located at C-25/26 from H_2_-26, H_3_-27, H_3_-28 to C-25, respectively; therefore, sinleptosterol B (**2**) was designated as structure **2**. The relative configurations at C-3, C-8, C-10, C-13, C-14, and C-17 of **2** were found to be the same as those of **1** in the core rings A–C. Note that the stereogenic carbons were identical to those of **1**, which were in agreement with the observed ^1^H and ^13^C NMR chemical shifts and proton coupling constants. Therefore, compound (**2**) was unambiguously identified as presented in Figure 1 and it was named sinleptosterol B.

Through a comparison of NMR spectroscopic data with those reported in the literature [5,12], the known compounds were identified as 8*α*H-3*β*,11-dihydroxy-24-methylene-9,11-secocholest-5-en-9-one (**3**), 8*β*H-3*β*,11-dihydroxy-24-methylene-9,11-secocholest-5-en-9-one (**4**), leptosterol A (**5**), (24*S*)-3*β*,11-dihydroxy-24-methyl-9,11-secocholest-5-en-9-one (**6**), and 3*β*,11-dihydroxy-9,11- secogorgost-5-en-9-one (**7**).

It is worth noting that the configurations of H-8 for compounds **3** and **4** were elucidated by comparing their ^1^H NMR data [12]. A large downfielding of the H-8 as well an upfielding of C-8 were identified to resonate from δ_H_ 2.69/δ_C_ 48.6 (compound **3**) to δ_H_ 3.02/δ_C_ 43.8 (compound **4**). Moreover, the ^1^H- and ^13^C-NMR spectroscopic features for positions 7 and 11 were also found to be different between these two compounds (**3**:δ_H_ 2.20 (H-7a, m) and δ_H_ 2.60 (H-7b, m)/δ_C_ 30.4 (C-7), 3.72 (H-11, 2H, t, *J* = 8.0 Hz)/δ_C_ 59.2 (C-11); **4**:δ_H_ 1.98 (H-7a, m) and δ_H_ 2.40 (H-7b, m)/δ_C_ 33.0 (C-7), δ_H_ 3.83 (H-11a, 1H, m) and δ_H_ 3.68 (H-11b, 1H, m)/δ_C_ 59.3 (C-11)) (Supplementary Materials Figures S19–S22).

### 2.3. Anti-Inflammatory Assessment of Isolated 9,11-Secosterols

The anti-inflammatory properties of metabolites **1**–**7** were characterized by assessing the inhibition of superoxide anion generation and elastase release by human neutrophils in response to fMLP/CB (Table 3). Compounds **1**–**5** were shown to inhibit superoxide anion generation and elastase release, at concentrations ranging from 1.63 to 8.07 μM. The IC_50_ values of compounds 3 and 4 were lower than the other isolates. Secosteroid **7** presented activity at a concentration of 10 μM, indicating that the unique gorgosterol side chain nullified the anti-inflammatory activities.

*N*-Formyl peptide receptors (FPRs) are a family of G-protein coupled receptors involved in the switching on of leucocyte responses during inflammation. Human FPR1 is expressed primarily in neutrophils, monocytes, and macrophages. It also initiates immune reactions in response to several formyl peptide ligands derived from bacteria or mitochondria [13]. Researchers have previously proven that compounds acting as FPR1 antagonists exhibit anti-inflammatory activity in vitro and in vivo [14,15,16].

**Figure 4:**
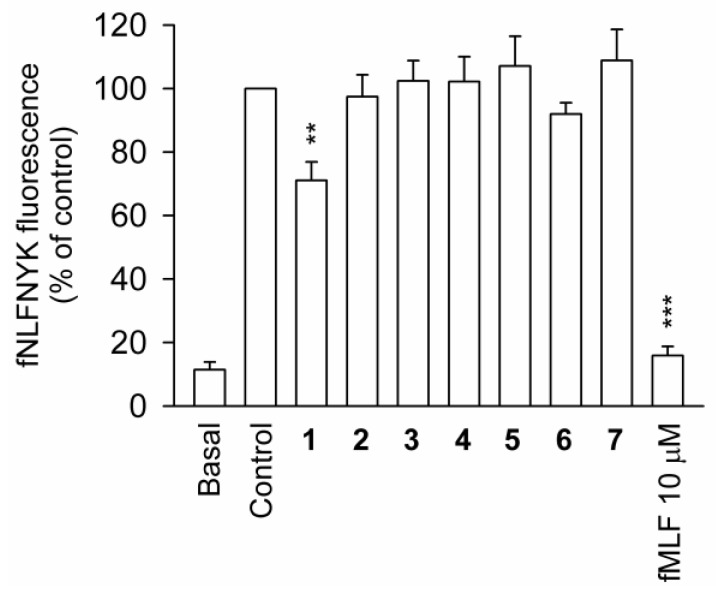
FPR1 receptor binding assay of compounds **1**–**7** in human neutrophils. Data are representative of three experiments. ** *p* < 0.01, *** *p* < 0.001 versus the control group.

Flow cytometry was used to determine whether compounds **1**–**7** possess binding affinity to FPR1, the receptor-binding assay of *N*-formyl-Nle-Leu-Phe-Nle-Tyr-Lys-fluorescein (fNLFNYK), an FPR1-specific fluorescent analog, on the surface of neutrophils. The results revealed that fMLF (10 μM) entirely inhibited the binding of fNLFNYK (2 nM), whereas only compound **2** presented a low affinity toward the FPR1 receptor at a concentration of 10 μM (Figure 4).

## 3. Experimental

### 3.1. General Procedures

The specific rotation of compounds was measured using a Jasco P-1010 digital polarimeter (Japan Spectroscopic Corporation, Tokyo, Japan). IR spectra were acquired using a Jasco FT/IR-4100 spectrometer (Japan Spectroscopic Corporation, Tokyo, Japan). NMR spectra were recorded on a Jeol Resonance ECZ400S NMR spectrometer (at 400 and 100 MHz for ^1^H and ^13^C NMR, respectively) using the residual CHCl_3_ signal (δ_H_ 7.26 ppm) as an international standard for ^1^H NMR and CDCl_3_ (δ_C_ 77.1 ppm) for ^13^C NMR, respectively. ESIMS and HRESIMS were recorded using a mass spectrometer (7 Tesla SolariX FTMS system; Bruker, Bremen, Germany and Xevo G2-XS QToF; Waters Corporation, Wilmslow, UK). Silica gel (230–400 mesh, Merck, Darmstadt, Germany) was used for open-column chromatography. Reverse-phase MPLC (RP-MPLC) was performed using a system comprising a gradient controller Model 601 (FLOM, Tokyo, Japan) and two Dual Pumps Model 204 (FLOM, Tokyo, Japan). Reversed-phase HPLC (RP-HPLC) was performed using a system comprising of Nexera-i LC-2040C 3D (Shimadzu, Kyoto, Japan) with photodiode-array (PDA) detector and a Rheodyne 7725i injection port (Rheodyne LLC., Rohnert Park, CA, USA). Post-run analysis was performed using Labsolutions 5.90. A Biotage^®^ SNAP Ultra C18 (12 g) flash cartridge (Biotage AB, Uppsala, Sweden) was used for RP-MPLC and a reverse phase column (COSMOSIL C18-AR-II, 10 × 250 mm, Nacalai Tesque, Inc., Kyoto, Japan) was used for RP-HPLC.

### 3.2. Coral Material

In November 2018, samples of the soft coral *Sinularia leptoclados* were obtained by hand using self-contained underwater breathing apparatus (SCUBA) off the coast of Pingtung, Taiwan. The samples were stored in a freezer at −20 °C until extraction. A specimen voucher was deposited with the Research Center for Chinese Herbal Medicine, Chang Gung University of Science and Technology, Taiwan (specimen No.: CGUST-C004-2018-NOV).

### 3.3. Extraction and Isolation

Soft coral material (wet weight 1742 g, dry weight 488 g) was cut into small pieces prior to ethyl acetate (EtOAc) extraction at room temperature. The EtOAc layer (9.6 g; superoxide anion generation: IC_50_ 3.97 μg/mL; elastase release: IC_50_ > 10 μg/mL) was separated on silica gel and eluted using *n*-hexane/EtOAc (stepwise, pure *n*-hexane−pure EtOAc−pure MeOH) to yield 22 fractions A−V. Fraction P (superoxide anion generation: IC_50_ 3.57 μg/mL; elastase release: IC_50_ 6.80 μg/mL) was further separated by RP-MPLC using a mixture of MeOH/ddH_2_O (*v*:*v* = 90:10 of volume ratio at a flow rate of 8.0 mL/min) to obtain compound **7** (82.5 mg) and nine subfractions, P1−P9. Subfraction P3 (superoxide anion generation: IC_50_ 2.06 μg/mL; elastase release: IC_50_ 1.45 μg/mL) was repurified by RP-HPLC using a mixture of acetonitrile/ddH_2_O (volume ratio of *v*:*v* = 90:10 at a flow rate of 2.0 mL/min) to derive **1** (2.6 mg), **2** (2.0 mg), **3** (3.5 mg), **4** (100.4 mg) and **5** (13.3 mg). Subfraction P4 was purified by RP-HPLC using a mixture of MeOH/ddH_2_O (volume ratio of *v*:*v* = 90:10 at a flow rate of 2.0 mL/min) to yield compound **6** (6.8 mg).

Sinleposterol A (**1**): colorless oil; [α${]}_{D}^{22}$ −16.31 (*c* 0.095, CHCl*_3_*); IR (neat) ν_max_ 3369, 2958, 1708 cm^−1^; ^1^H (400 MHz, CDCl_3_) and ^13^C (100 MHz, CDCl_3_) NMR data (see Table 1); ESIMS: *m*/*z* 451 [M + Na]^+^; HRESIMS: *m*/*z* 451.31811 (calcd. for C_28_H_44_O_3_ + Na, 451.31827).

Sinleposterol B (**2**): colorless oil; [α${]}_{D}^{24}$ −36 (*c* 0.1, CHCl_3_); IR (neat) ν_max_ 3406, 2954, 1708 cm^−1^; ^1^H (400 MHz, CDCl_3_) and ^13^C (100 MHz, CDCl_3_) NMR data (see Table 2); ESIMS: *m*/*z* 451 [M + Na]^+^; HRESIMS: *m*/*z* 451.3239 (calcd. for C_28_H_44_O_3_ + Na, 451.3188). 

### 3.4. In Vitro Anti-Inflammatory Bioassay

#### 3.4.1. Human Neutrophil Superoxide Anion Generation and Elastase Release

Blood samples were collected from healthy adult donors (20–32 years) via venipuncture in accordance with the standard protocol approved by the local institutional review board. Human neutrophils were isolated from peripheral blood through dextran sedimentation, centrifugation in a Ficoll–Hypaque gradient, and hypotonic lysis of red blood cells. In accordance with the above mentioned protocol, assays were performed to measure superoxide anion generation based on the superoxide dismutase-inhibitable reduction of ferricytochrome C. Note that methoxy-succinyl-alanyl-alanyl-prolyl-valine-*p*-nitroanilide (MeO-Suc-Ala-Ala-Pro-Val-*p*-nitroanilide) was used as the enzyme substrate for the detection of elastase release [17,18]. 

#### 3.4.2. Receptor Binding Assay

Receptor binding assays were performed via FACScan analysis of fNLFNYK binding, a fluorescent analog of fMLF, as described in [16]. Neutrophils, differentiated THP-1, or FPR1-expressed HEK-293 were preincubated with test compounds at 4 °C for 5 min and then labelled with fNLFNYK for 30 min. Cells were immediately analyzed via flow cytometry.

#### 3.4.3. Statistical Analysis

All experiments were conducted at least three times and the results are presented as the mean ± SEM. Statistical analysis was performed using the Student’s *t*-test, and a *p* value < 0.05 was considered statistically significant. Sigma Plot software (version 8.0, Systat Software, San Jose, CA, USA) was used for all statistical analysis [17]. 

## 4. Conclusions

Marine 9,11-secosteroid was first isolated from the gorgonian *Pseudopterogorgia americana* in 1972 [19]. Research since that time has revealed a variety of compounds in this group from a variety of invertebrates, including sponges, gorgonians, octocorals, ascidian, mollusk, and sea hare [7,20,21]. In recent years, 9,11-secosteroids have attracted considerable attention for their anti-inflammatory and antihepafibrosis properties [9,11,22,23,24,25]. This study strategically isolated two novel metabolites, sinleptosterols A and B, and five known compounds from the soft coral *Sinularia leptoclados*. The effectiveness of compounds 1–7 in inhibiting fMLP/CB-induced neutrophilic inflammation was also assessed. Compound 3 and 4 presented the most potent anti-inflammatory activities in terms of superoxide anion production and elastase emancipation. Cell free binding assays also demonstrated that the anti-inflammatory properties of these 9,11-secosteroids did not follow the pathway of conventional FPR1 antagonists.

## Figures and Tables

**Table 1:** ^1^H (400 MHz, CDCl_3_) and ^13^C (100 MHz, CDCl_3_) NMR data and COSY and HMBC for **1**.

Position	δ_H_ (*J* in Hz)	δ_C_, Type	COSY	HMBC
1a/b	1.50 m; 1.81 m	31.0, CH_2_	H_2_-2	C-3
2a/b	1.40 m; 1.93 m	30.8, CH_2_	H_2_-1, H-3	n. o. ^a^
3	3.51 m	71.4, CH	H_2_-2, H_2_-4	n. o.
4a/b	2.23 m; 2.44 m	40.6, CH_2_	H-3	C-3, C-5, C-6
5	-	140.4, C	-	-
6	5.47 brd (5.6)	121.5, CH	H_2_-7	C-4, C-7, C-8, C-10
7a/b	1.98 m; 2.40 m	33.1, CH_2_	H-6, H-8	C-5, C-6
8	3.03 ddd (12.4, 12.4, 6.8)	43.8, CH	H_2_-7, H-14	C-7, C-9, C-14
9	-	217.5, C	-	-
10	-	48.4, C	-	-
11a/b	3.86 m; 3.74m	59.4, CH_2_	H_2_-12	n. o.
12	1.33 m; 1.67 m	40.2, CH_2_	H_2_-11	C-11, C-13
13	-	45.5, C	-	-
14	2.61 m	42.1, CH	H-8, H_2_-15	C-9
15	1.31 m; 1.58 m	24.6, CH_2_	H-14, H_2_-16	C-14
16	1.31 m; 1.71 m	24.8, CH_2_	H_2_-15, H-17	n. o.
17	1.78 m	49.6, CH	H_2_-16, H-20	n. o.
18	0.69 s	17.7, CH_3_	-	C-12, C-13, C-14, C-17
19	1.40 s	22.8, CH_3_	-	C-1, C-5, C-9, C-10
20	2.25 m	38.5, CH	H-17, H_3_-21, H-22	C-16
21	1.08 d (6.4)	22.1, CH_3_	H-20	C-17, C-20
22	5.65 dd (16.0, 7.2)	134.6, CH	H-20, H-23	C-20, C-21, C-24
23	5.93 d (16.0)	130.2, CH	H-22	C-20, C-24, C-25, C-28
24	-	153.0, C		-
25	2.54 m	29.4, CH	H_3_-26, H_3_-27	C-24, C-28
26	1.06 d (6.8)	22.4, CH_3_	H-25	C-24, C-25
27	1.07 d (6.8)	21.5, CH_3_	H-25	C-24, C-25
28	4.83 d (6.8)	109.8, CH_2_		C-24, C-25

^a^ n. o. = not observed.

**Table 2:** ^1^H (400 MHz, CDCl_3_) and ^13^C (100 MHz, CDCl_3_) NMR data and COSY and HMBC for **2**.

Position	δ_H_ (*J* in Hz)	δ_C_, Type	COSY	HMBC
1a/b	1.53 m; 1.90 m	30.8, CH_2_	H_2_-2	n. o. ^a^
2a/b	1.31 m; 1.93 m	31.3, CH_2_	H_2_-1, H-3	n. o.
3	3.51 m	71.4, CH	H_2_-2, H_2_-4	n. o.
4a/b	2.23 m; 2.44 m	40.6, CH_2_	H-3	n. o.
5	-	140.4, C	-	-
6	5.48 brd (5.6)	121.5, CH	H_2_-7	C-4, C-7, C-8
7a/b	2.02 m; 2.39 m	33.0, CH_2_	H-6, H-8	n. o.
8	3.03 td (12.4, 6.8)	43.6, CH	H_2_-7, H-14	C-7, C-9, C-14
9	-	217.5, C	-	-
10	-	48.5, C	-	-
11a/b	3.69 m; 3.83m	59.4, CH_2_	H_2_-12	n. o.
12	1.32 m; 1.68 m	40.3, CH_2_	H_2_-11	n. o.
13	-	45.6, C	-	-
14	2.61 m	41.8, CH	H-8, H_2_-15	n. o.
15	1.30 m; 1.56 m	24.4, CH_2_	H-14, H_2_-16	n. o.
16	1.30 m; 1.74 m	25.0, CH_2_	H_2_-15, H-17	-
17	1.65 m	49.2, CH	H_2_-16	-
18	0.67 s	17.3, CH_3_	-	C-11, C-12, C-13, C-14, C-17
19	1.38 s	22.9, CH_3_	-	C-1, C-5, C-9, C-10
20	1.41 m	34.0, CH	H_3_-21	n. o.
21	1.01 d (6.8)	19.4, CH_3_	H-20	C-17, C-20, C-22
22	1.17 m; 1.51 m	34.7, CH_2_	H-23	n. o.
23	1.81 m; 2.39 m	31.0, CH_2_	H-22	n. o.
24	-	148.5, C	-	
25	-	142.7, C	-	
26	4.59 d (1.2)	112.5, CH_2_	-	C-25
27	1.90 s	21.2, CH_3_	-	C-24, C-25, C-26
28	5.06 s	112.0, CH_2_	-	C-23, C-24, C-25

^a^ n. o. = not observed.

**Table 3:** Effects of compounds **1**–**7** on superoxide anion generation and elastase release in fMLF/CB-induced human neutrophils.

Compound	Superoxide Anions		Elastase Release	
	IC_50_ (μM) ^a^	Inh %	IC_50_ (μM)	Inh %
**1**	7.07 ± 0.52	64.76 ± 3.42 ***	7.57 ± 0.40	65.04 ± 2.76 ***
**2**	4.68 ± 0.57	76.30 ± 5.09 ***	4.29 ± 0.25	105.09 ± 5.25 ***
**3**	1.97 ± 0.12	90.47 ± 2.44 ***	3.12 ± 0.07	112.23 ±5.01 ***
**4**	2.96 ± 0.91	91.11 ± 4.51 ***	1.63 ± 0.15	93.74 ± 1.23 ***
**5**	8.07 ± 0.53	57.93 ± 2.30 ***	4.73 ± 0.57	86.32 ± 2.91 ***
**6**	4.09 ± 0.50	60.51 ± 4.06 ***	>10	25.38 ± 6.68 **
**7**	>10	10.29 ± 5.42 ***	>10	18.87 ± 3.86 ***

Percentage of inhibition (Inh %) at 10 μM concentration. Results are presented as mean ± S.E.M. (*n* = 3 or 4). ** *p* < 0.01, *** *p* < 0.001 compared with the control (DMSO). ^a^ Concentration necessary for 50% inhibition (IC_50_).

## Supplementary Materials

The following are available online at https://www.mdpi.com/1660-3397/18/5/271/s1. HRESIMS, IR, 1D (^1^H NMR, ^13^C NMR, and DEPT spectra), and 2D (COSY, HSQC, HMBC, and NOESY) NMR spectra of new compounds **1** and **2**. ^1^H NMR and ^13^C NMR spectra of compounds **3** and **4**.
