# Supplementary material for: 1H NMR-Based Isolation of Anti-Inflammatory 9,11-Secosteroids from the Octocoral Sinularia leptoclados
Source: Mar Drugs. 2020 May 21;18(5):271. doi: 10.3390/md18050271 (PMC7281107; doi:10.3390/md18050271)
Supplement: Supplementary file 1 [file marinedrugs-18-00271-s001.pdf]

Supporting Material:

^{1}H NMR-based isolation of anti-inflammatory 9,11-secosteroids from the octocoral *Sinularia leptoclados*

Yu-Chia Chang [1], Kuei-Hung Lai [1], Sunil Kumar [2], Po-Jen Chen [3], Yi-Hsuan Wu [1], Ching-Long Lai [1,4], Hsi-Lung Hsieh[1,4,5,*], Ping-Jyun Sung [6,7,8,*], and Tsong-Long Hwang [1,2,9,10,11,*]

[1] Research Center for Chinese Herbal Medicine, Graduate Institute of Healthy Industry Technology, College of Human Ecology, Chang Gung University of Science and Technology, Taoyuan 333324, Taiwan; jay0404@gmail.com (Y.-C.C.); mos19880822@gmail.com (K.-H.L.); yhwu03@mail.cgust.edu.tw (Y.-H.W.); dinolai@mail.cgust.edu.tw (C.-L.L.)
[2] Chinese Herbal Medicine Research Team, Healthy Aging Research Center, Chang Gung University, Taoyuan 333323, Taiwan; suniliftm1982@gmail.com
[3] Department of Cosmetic Science, Providence University, Taichung 433303, Taiwan; litlep@hotmail.com
[4] Department of Nursing, Division of Basic Medical Sciences, Chang Gung University of Science and Technology, Taoyuan 333324, Taiwan
[5] Department of Neurology, Chang Gung Memorial Hospital, Taoyuan 333423, Taiwan
[6] National Museum of Marine Biology and Aquarium, Pingtung 944401, Taiwan
[7] Graduate Institute of Marine Biology, National Dong Hwa University, Pingtung 944401, Taiwan
[8] Chinese Medicine Research and Development Center, China Medical University Hospital, Taichung 404394, Taiwan
[9] Graduate Institute of Natural Products, College of Medicine, Chang Gung University, Taoyuan 333323, Taiwan
[10] Department of Anaesthesiology, Chang Gung Memorial Hospital, Taoyuan 333423, Taiwan
[11] Department of Chemical Engineering, Ming Chi University of Technology, New Taipei City 243303, Taiwan

* Correspondence: hlhsieh@mail.cgust.edu.tw (H.-L.H.); pjsung@nmmba.gov.tw (P.-J.S.); htl@mail.cgu.edu.tw (T.-L.H.); Tel.: +886-3-211-8999 (ext. 5421) (H.-L.H.); +886-8-882-5037 (P.-J.S.); +886-3-211-8800 (T.-L.H.); Fax: +886-8-882-5087 (P.-J.S.); +886-3-211-8506 (T.-L.H.)

No	Content	page
**Figure S1.**	HRESIMS spectrum of compound **1**	3
**Figure S2.**	IR spectrum of compound **1**	3
**Figure S3.**	^{1}H NMR spectrum (400 MHz) of compound **1** in $CDCl_3$	4
**Figure S4.**	^{13}C NMR spectrum (100 MHz) of compound **1** in $CDCl_3$	4
**Figure S5.**	DEPT spectrum of compound **1** in $CDCl_3$	5
**Figure S6.**	gHSQC spectrum (400 MHz) of compound **1** in $CDCl_3$	5
**Figure S7.**	^{1}H–^{1}H COSY spectrum (400 MHz) of compound **1** in $CDCl_3$	6
**Figure S8.**	gHMBC spectrum (400 MHz) of compound **1** in $CDCl_3$	6
**Figure S9.**	NOESY spectrum (400 MHz) of compound **1** in $CDCl_3$	7
**Figure S10.**	HRESIMS spectrum of compound **2**	7
**Figure S11.**	IR spectrum of compound **2**	8
**Figure S12.**	^{1}H NMR spectrum (400 MHz) of compound **2** in $CDCl_3$	8
**Figure S13.**	^{13}C NMR spectrum (100 MHz) of compound **2** in $CDCl_3$	9
**Figure S14.**	DEPT spectrum of compound **1** in $CDCl_3$.	9
**Figure S15.**	gHSQC spectrum (400 MHz) of compound **2** in $CDCl_3$	10
**Figure S16.**	^{1}H–^{1}H COSY spectrum (400 MHz) of compound **2** in $CDCl_3$	10
**Figure S17.**	gHMBC spectrum (400 MHz) of compound **2** in $CDCl_3$	11
**Figure S18.**	NOESY spectrum (400 MHz) of compound **2** in $CDCl_3$	11
**Figure S19.**	^{1}H NMR spectrum (400 MHz) of compound **3** in $CDCl_3$	12
**Figure S20.**	^{13}C NMR spectrum (100 MHz) of compound **3** in $CDCl_3$	12
**Figure S21.**	^{1}H NMR spectrum (400 MHz) of compound **4** in $CDCl_3$	13
**Figure S22.**	^{13}C NMR spectrum (100 MHz) of compound **4** in $CDCl_3$	13

Mass Spectrum SmartFormula Report

Analysis Info

Analysis Name	D:\Data\a2\CGUSTCOO4P3C_000003.d	5/28/2019 4:52:36 PM
Method	broadband first signal	Operator: YU HSIAO-CHING
Sample Name	Cgust-c004p3c	Instrument: BRUKER FT-MS solariX
Comment	ESI Positive	

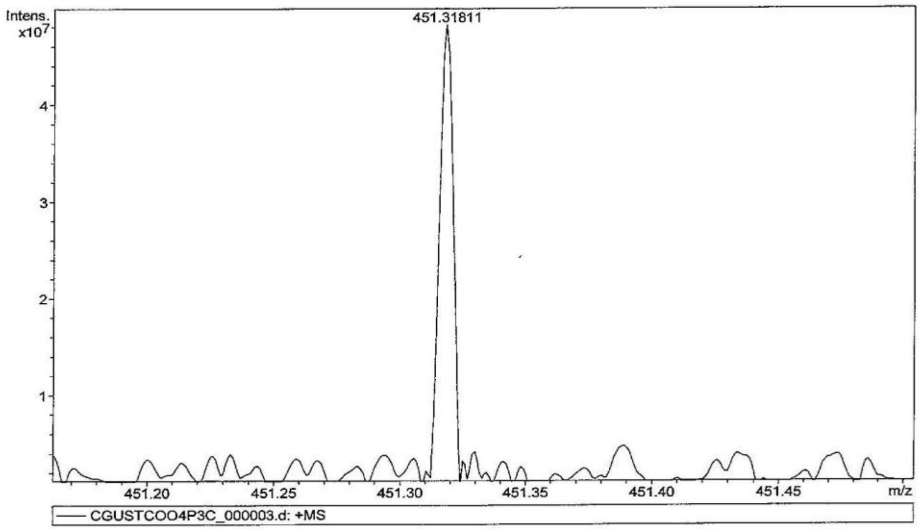

Meas. m/z	#	Formula	Score	m/z	err [mDa]	err [ppm]	mSigma	rdb	e⁻ Conf	N-Rule
451.31811	1	C 28 H 44 Na O 3	100.00	451.31827	0.15	0.34	27.3	6.5	even	ok

S1. HRESIMS spectrum of compound **1**

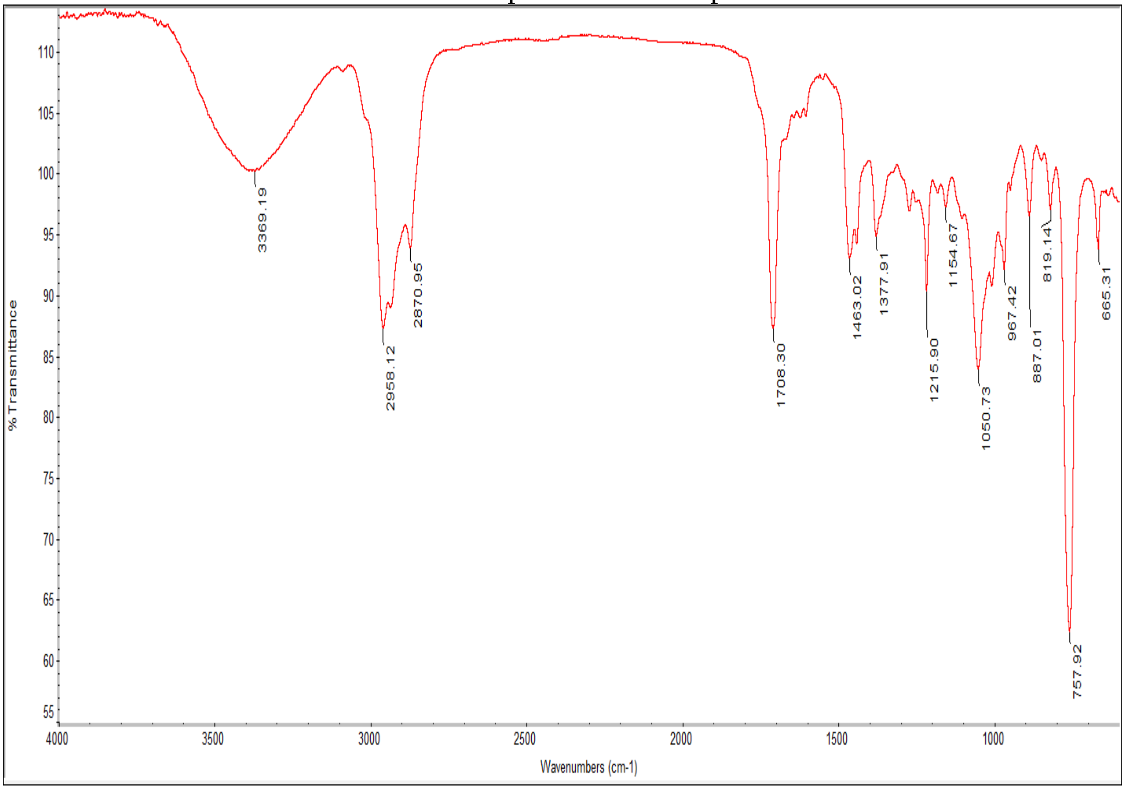

S2. IR spectrum of compound **1**

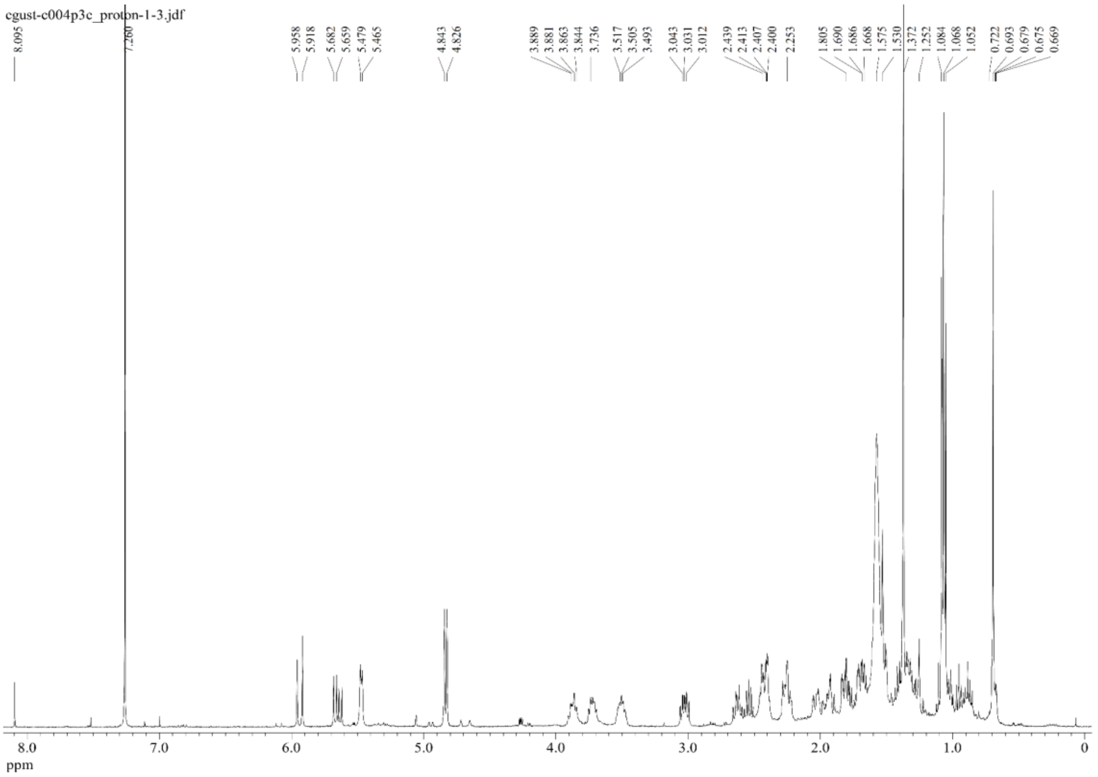

S3. ^{1}H NMR spectrum (400 MHz) of compound **1** in $CDCl_3$

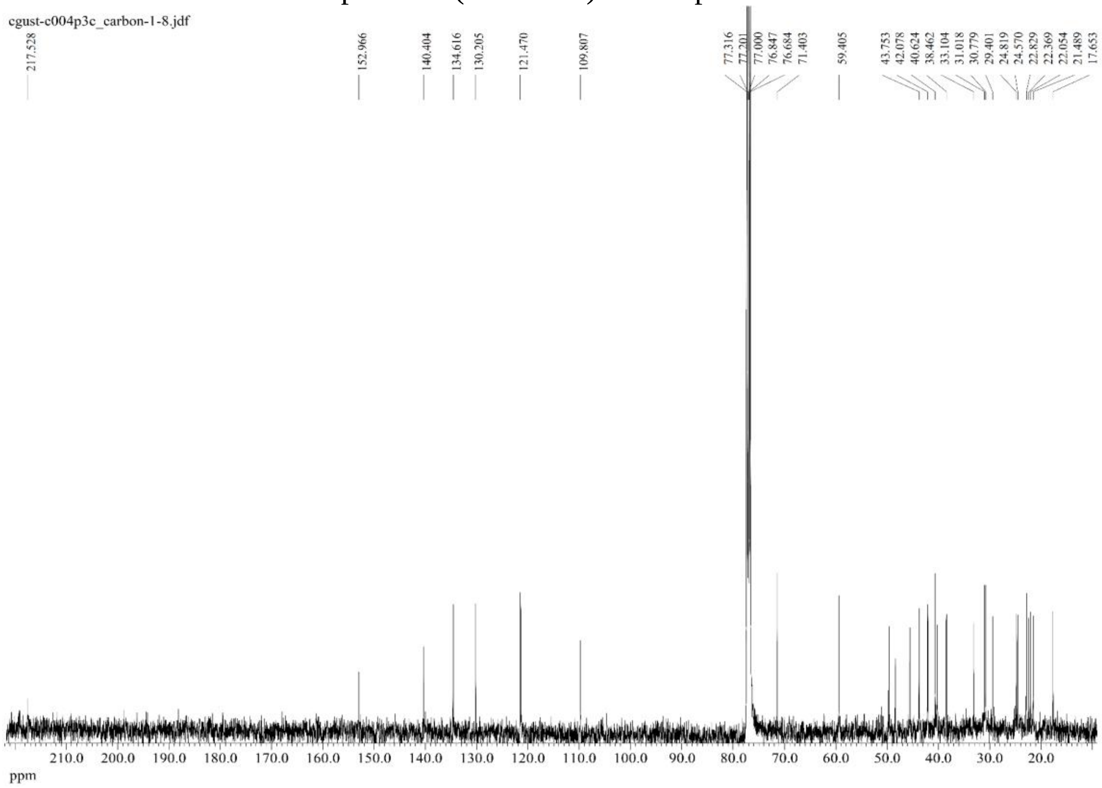

S4. ^{13}C NMR spectrum (100 MHz) of compound **1** in $CDCl_3$

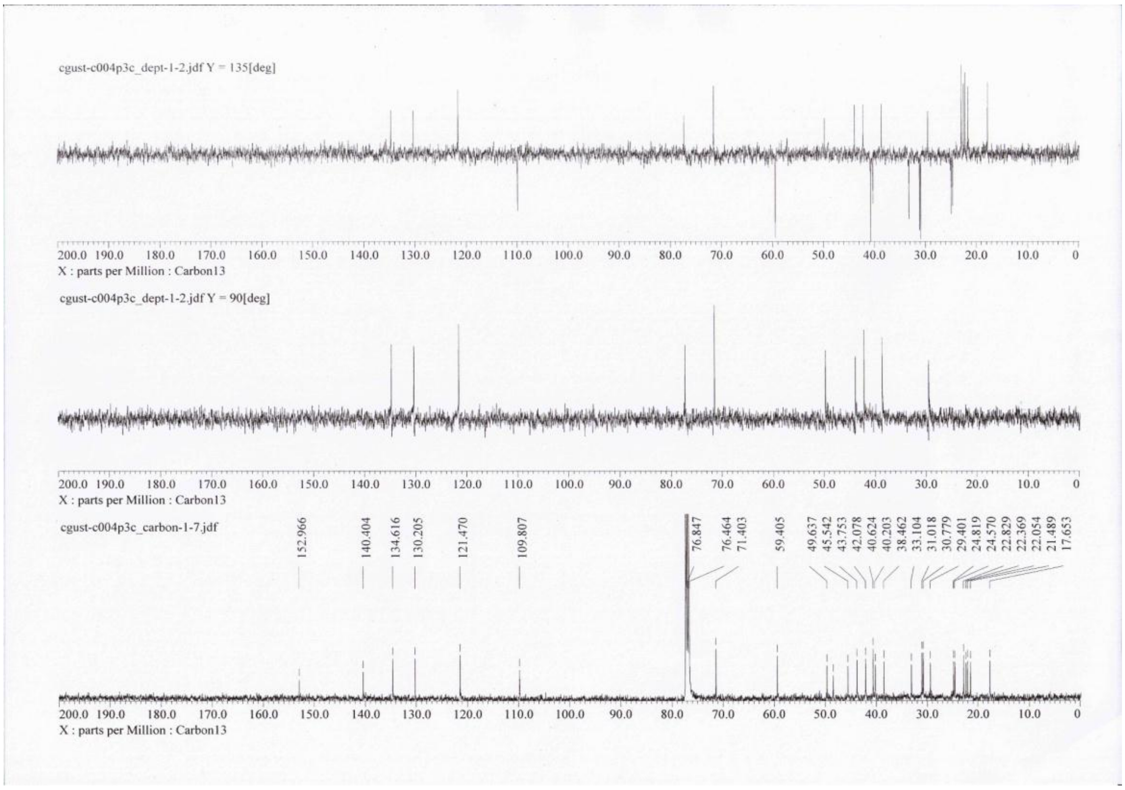

S5. DEPT spectrum of compound **1** in $CDCl_3$

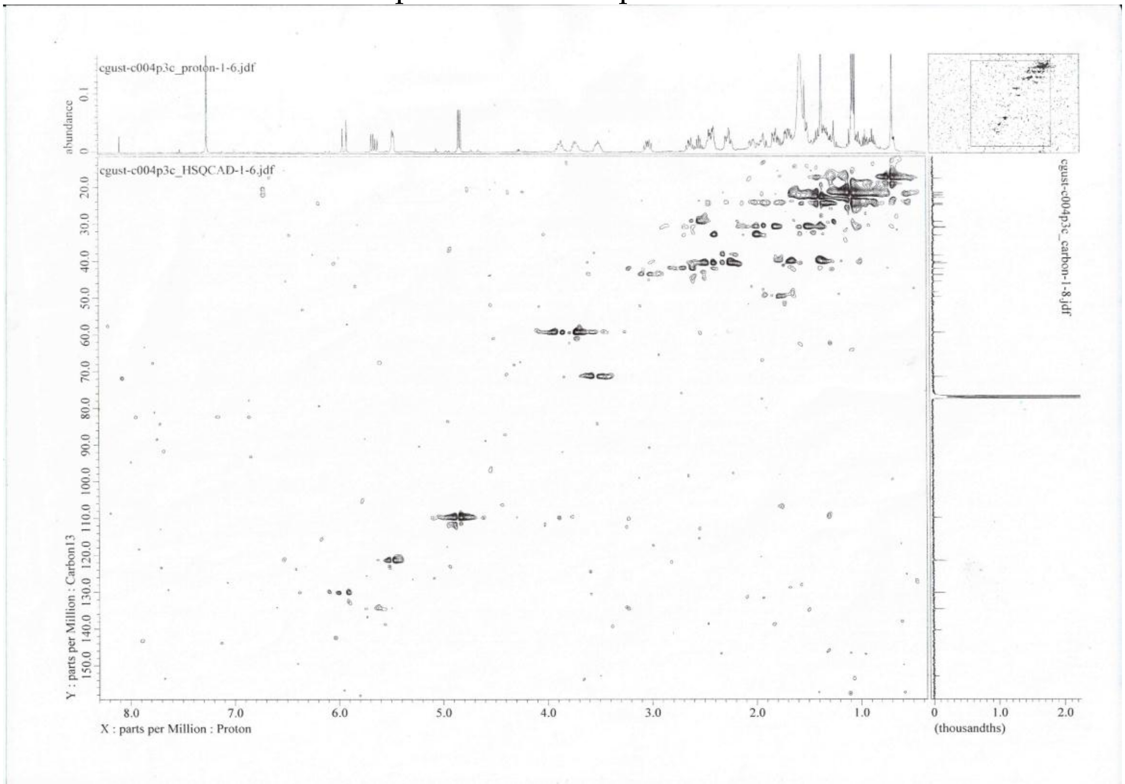

S6. gHSQC spectrum (400 MHz) of compound **1** in $CDCl_3$

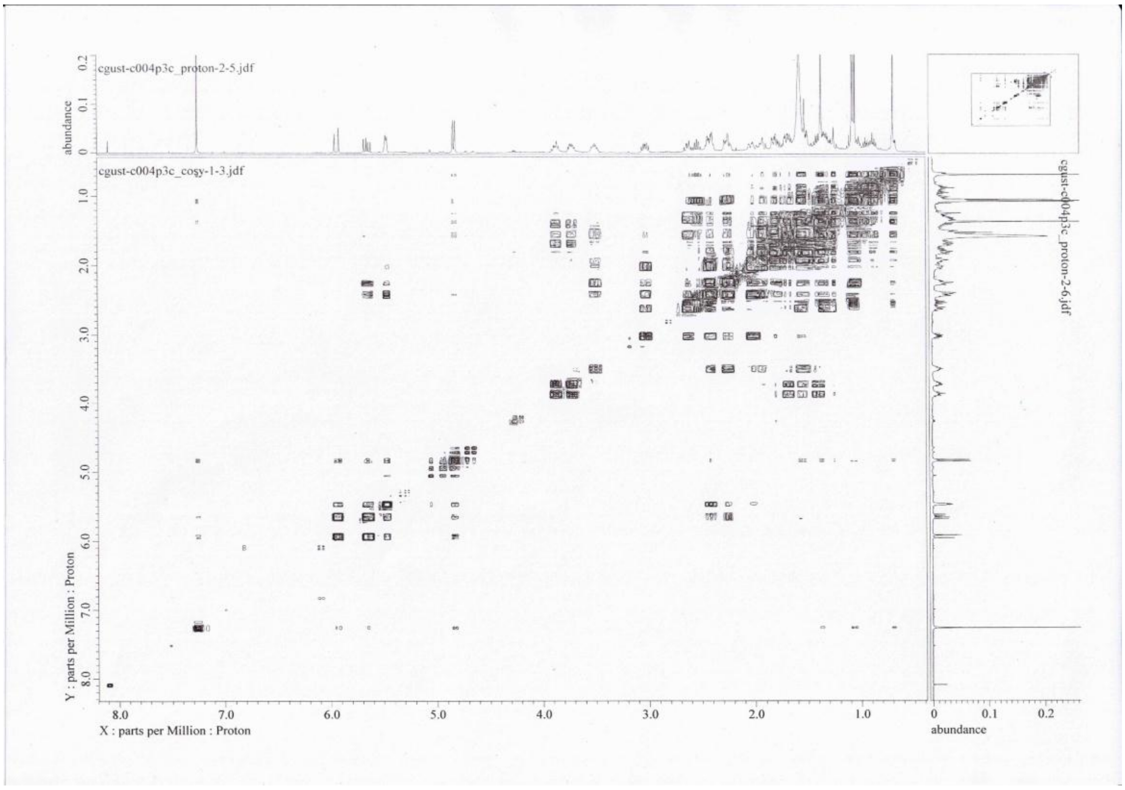

S7. ^{1}H–^{1}H COSY spectrum (400 MHz) of compound **1** in $CDCl_3$

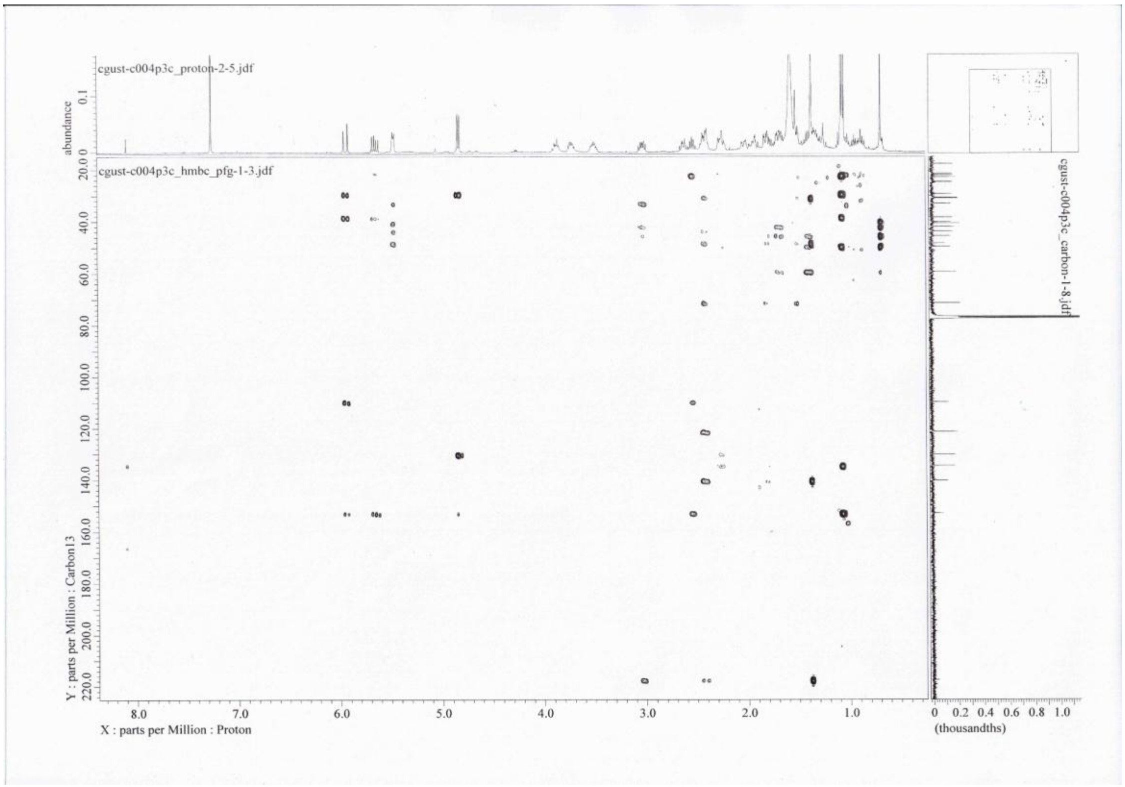

S8. gHMBC spectrum (400 MHz) of compound **1** in $CDCl_3$

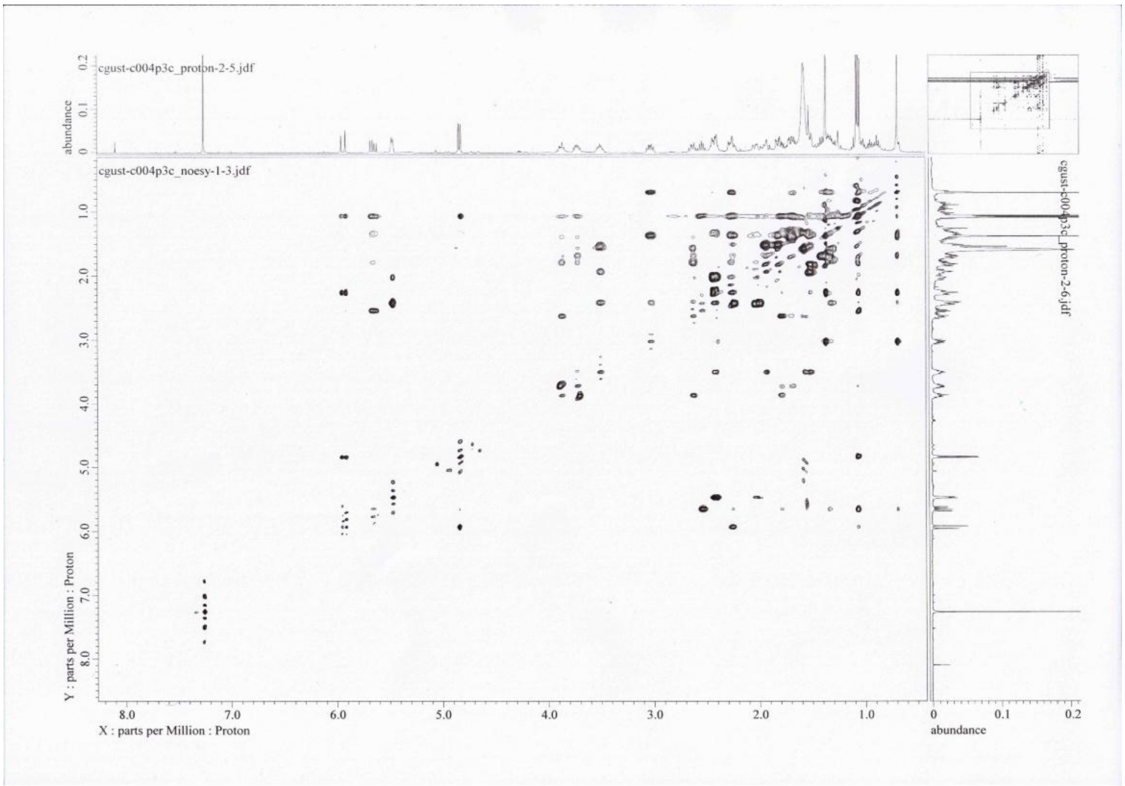

S9. NOESY spectrum (400 MHz) of compound **1** in $CDCl_3$

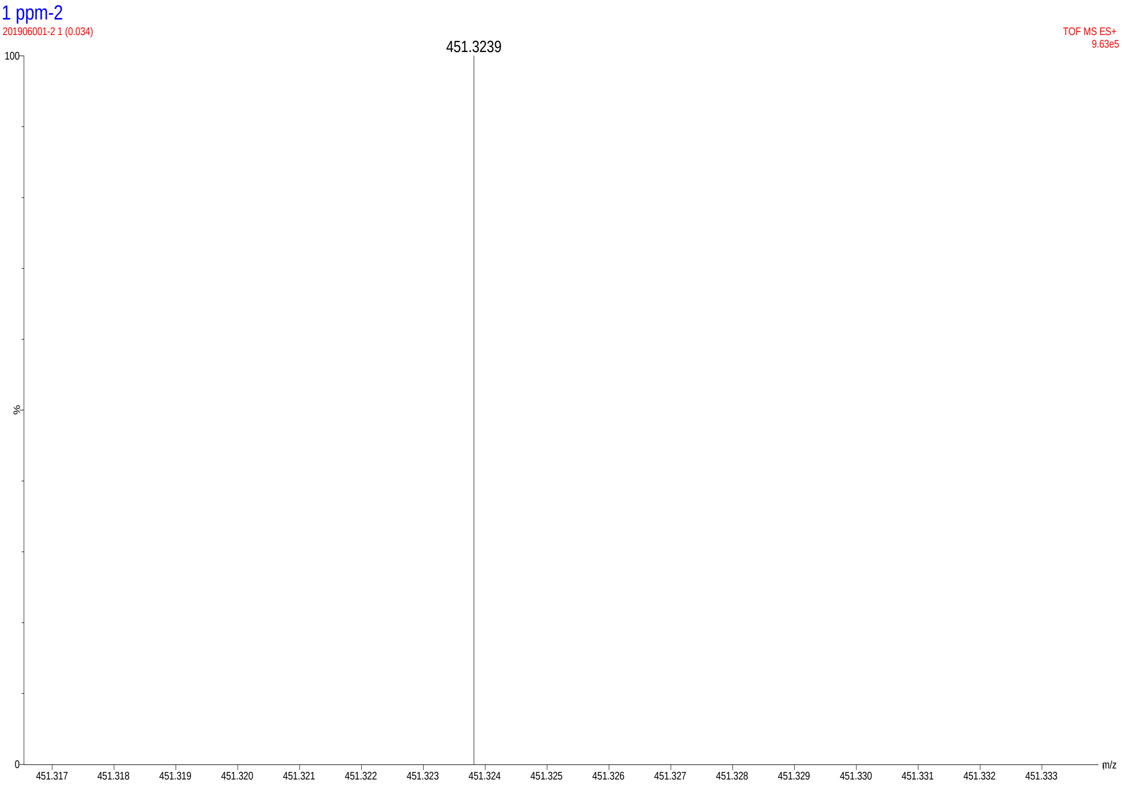

S10. HRESIMS spectrum of compound **2**

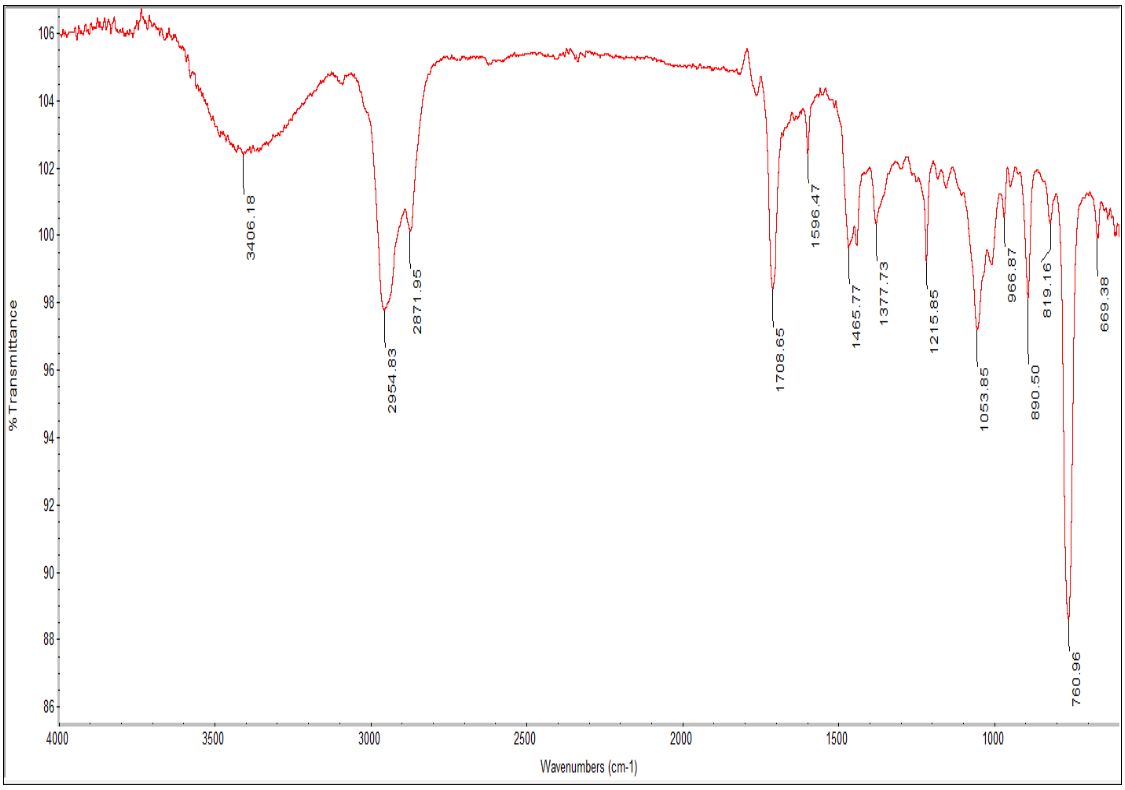

S11. IR spectrum of compound **2**

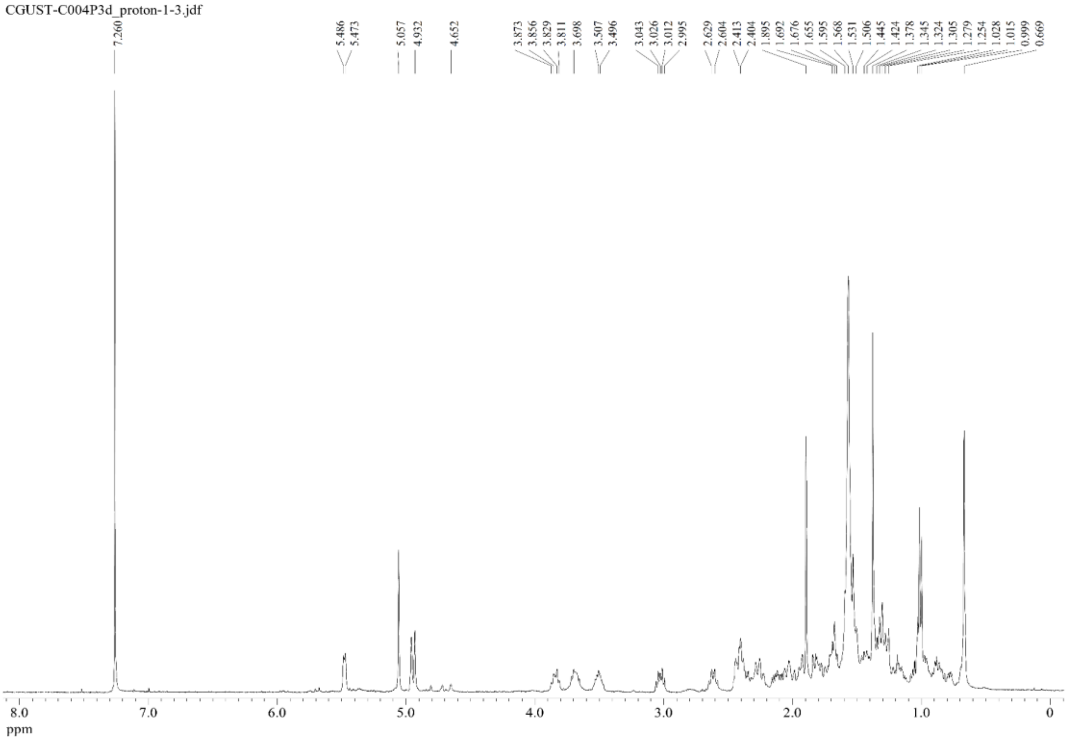

S12. ^{1}H NMR spectrum (400 MHz) of compound **2** in $CDCl_3$

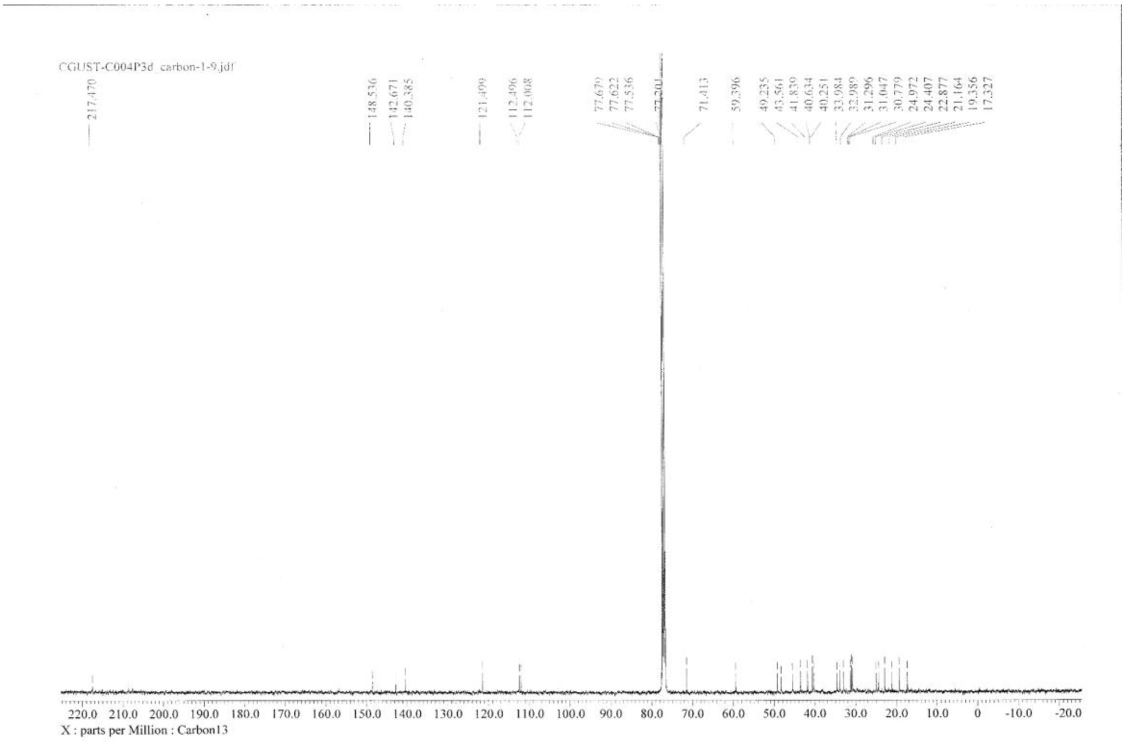

S13. ^{13}C NMR spectrum (100 MHz) of compound **2** in $CDCl_3$

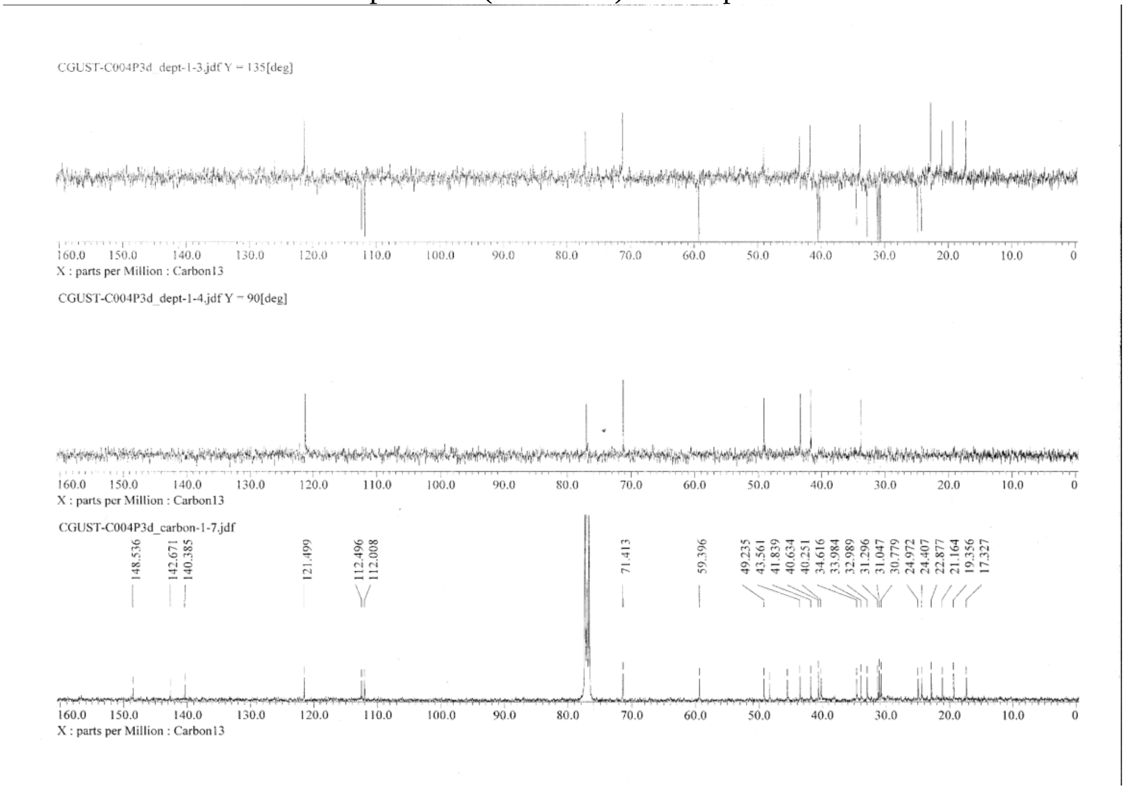

S14. DEPT spectrum of compound **2** in $CDCl_3$.

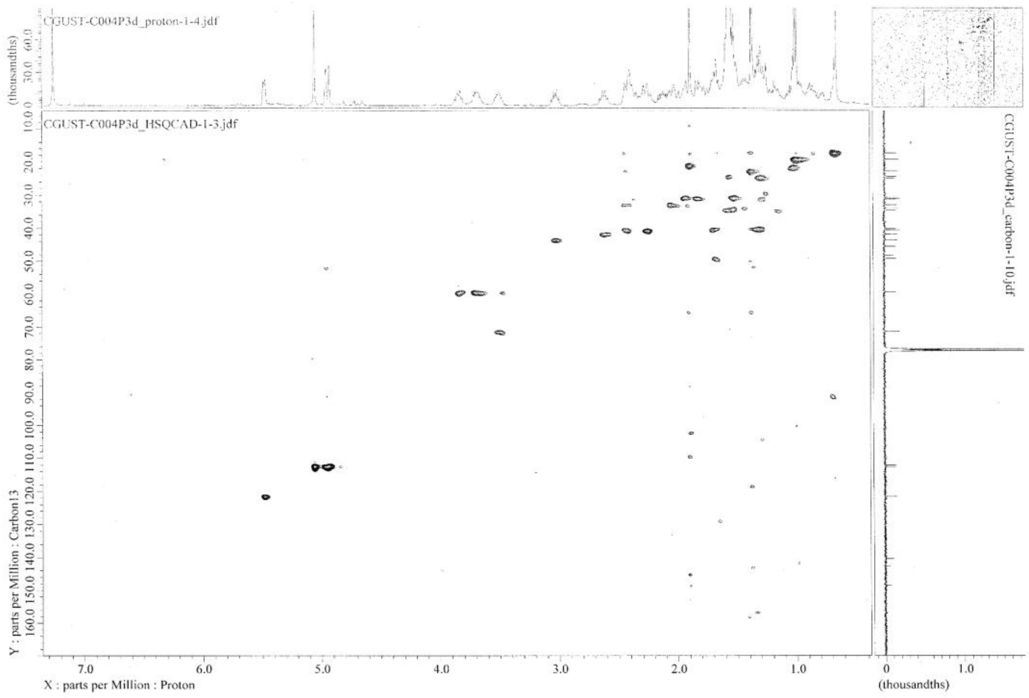

S15. gHSQC spectrum (400 MHz) of compound **2** in $CDCl_3$

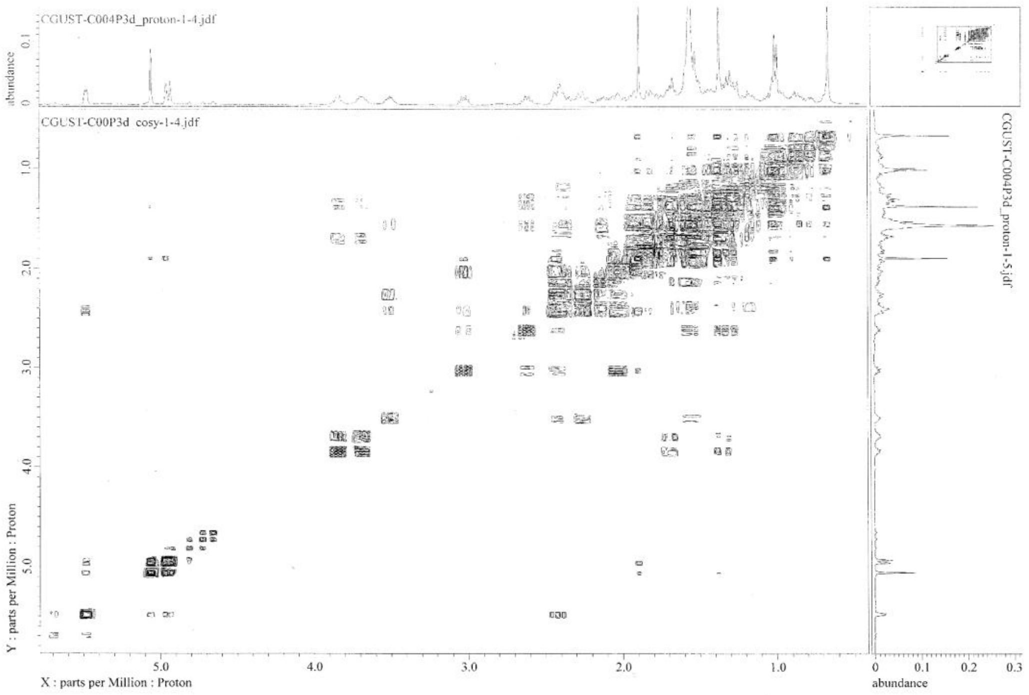

S16. ^{1}H–^{1}H COSY spectrum (400 MHz) of compound **2** in $CDCl_3$

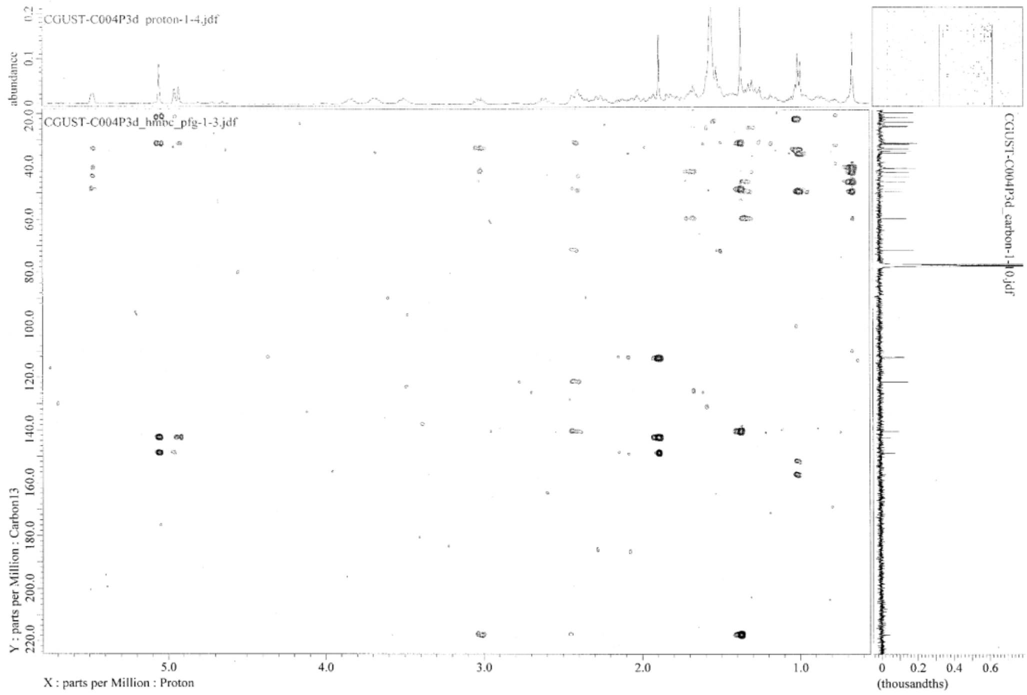

S17. gHMBC spectrum (400 MHz) of compound **2** in $CDCl_3$

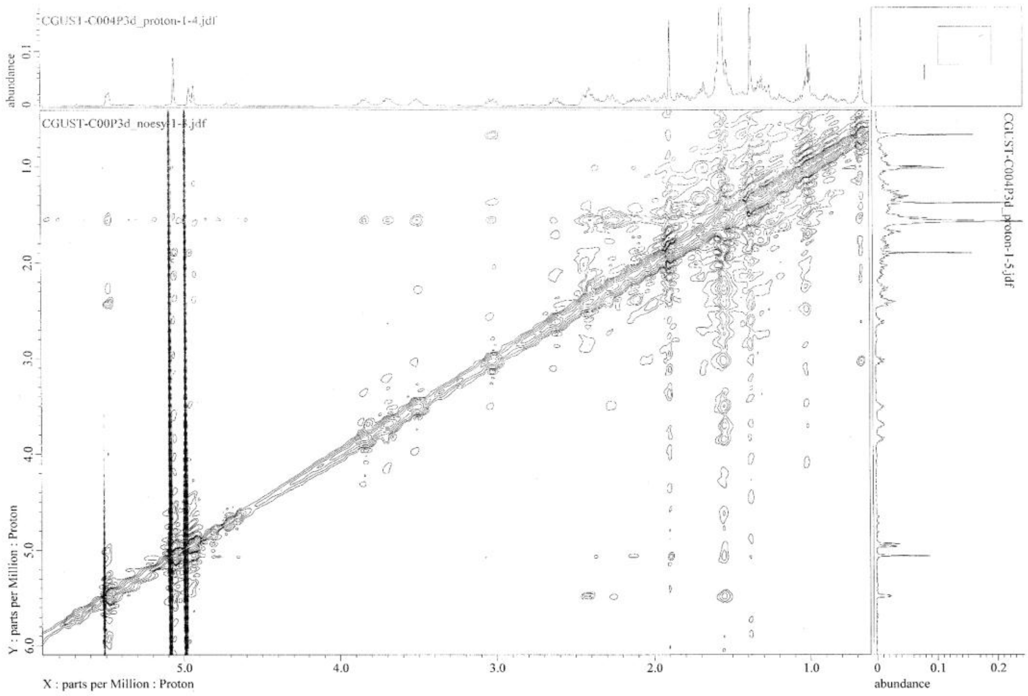

S18. NOESY spectrum (400 MHz) of compound **2** in $CDCl_3$

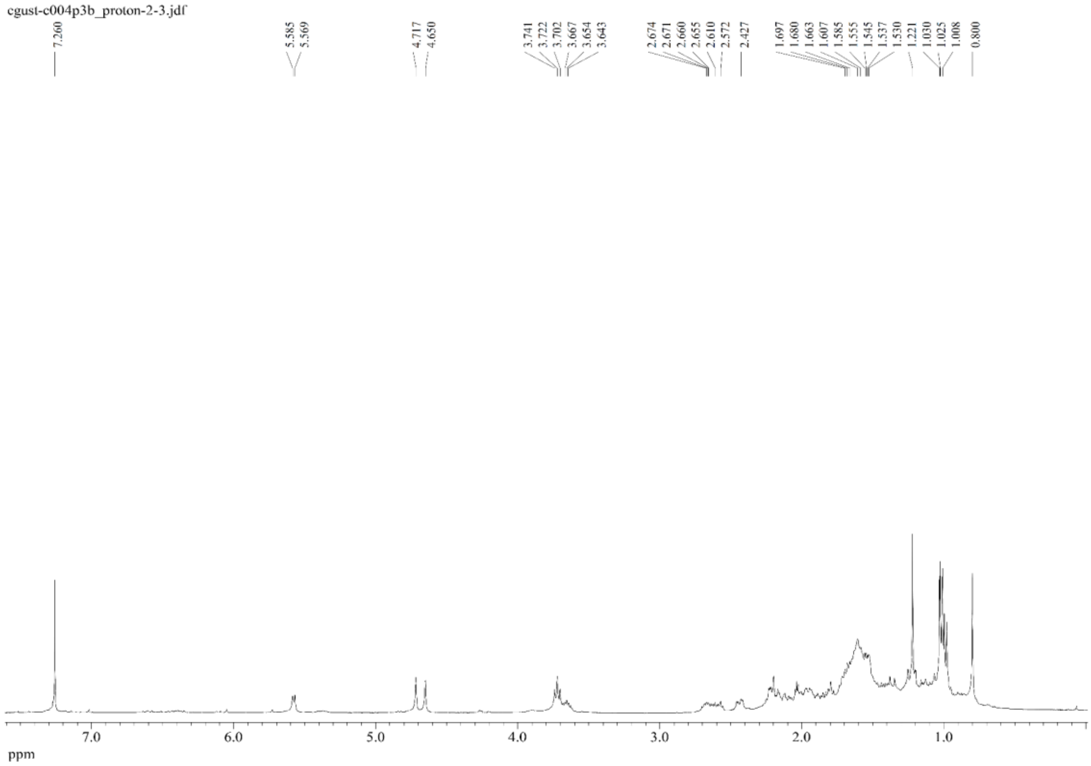

S19. ^{1}H NMR spectrum (400 MHz) of compound **3** in $CDCl_3$

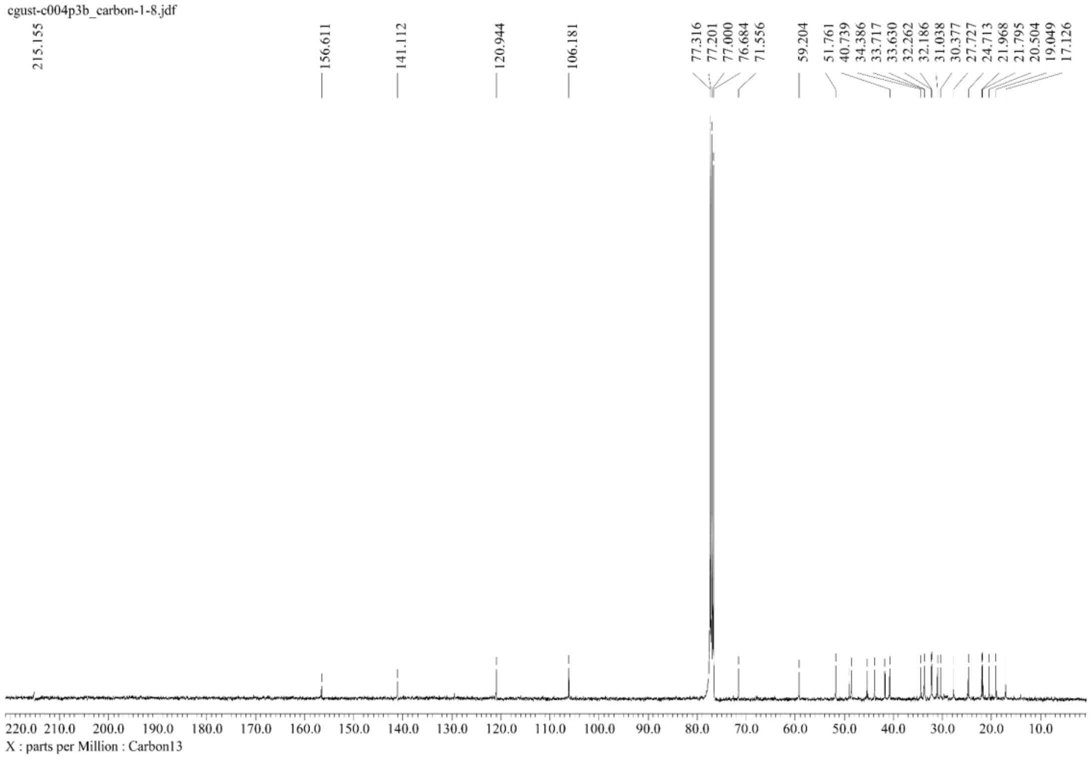

S20. ^{13}C NMR spectrum (100 MHz) of compound **3** in $CDCl_3$

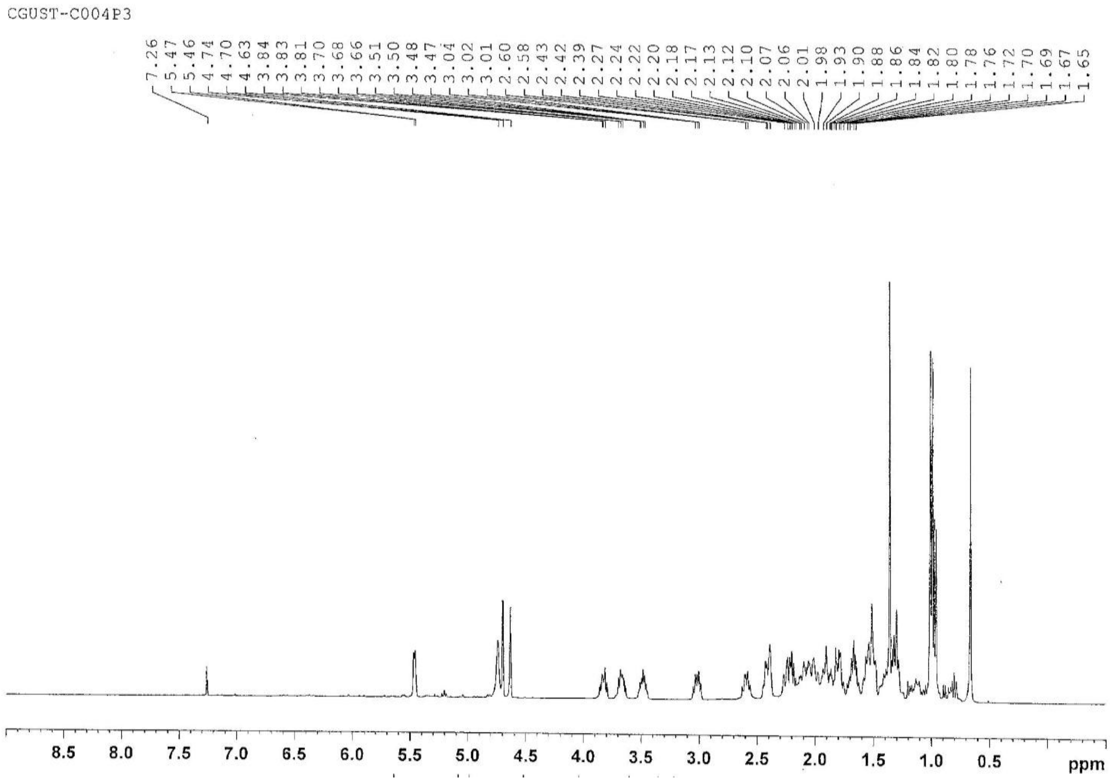

S21. ^{1}H NMR spectrum (400 MHz) of compound **4** in $CDCl_3$

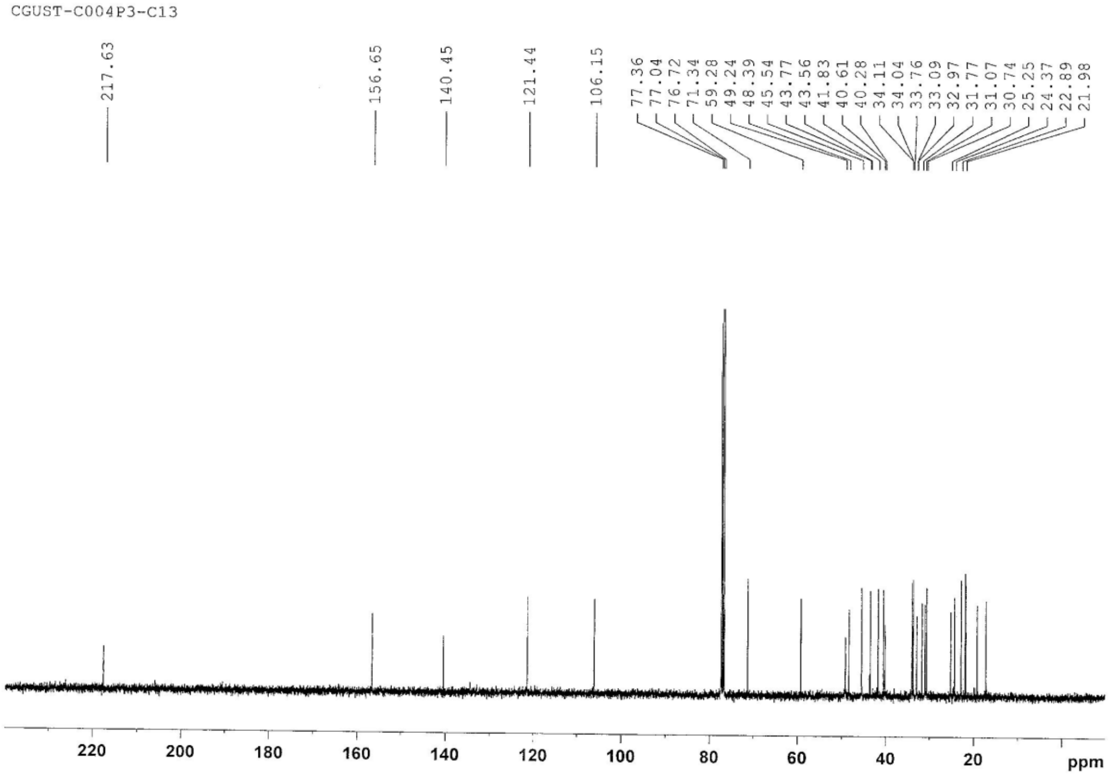

S22. ^{13}C NMR spectrum (100 MHz) of compound **4** in $CDCl_3$
